# Metal oxide nanostructures: preparation, characterization and functional applications as chemical sensors

**DOI:** 10.3762/bjnano.8.122

**Published:** 2017-06-06

**Authors:** Dario Zappa, Angela Bertuna, Elisabetta Comini, Navpreet Kaur, Nicola Poli, Veronica Sberveglieri, Giorgio Sberveglieri

**Affiliations:** 1 SENSOR, Dipartimento di Ingegneria dell’Informazione, Università degli Studi di Brescia and CNR-INO, via Valotti 9, 25123 Brescia, Italy

**Keywords:** chemical sensors, electronic nose, metal oxides, nanowires

## Abstract

Preparation and characterization of different metal oxide (NiO, WO_3_, ZnO, SnO_2_ and Nb_2_O_5_) nanostructures for chemical sensing are presented. p-Type (NiO) and n-type (WO_3_, SnO_2_, ZnO and Nb_2_O_5_) metal oxide nanostructures were grown on alumina substrates using evaporation–condensation, thermal oxidation and hydrothermal techniques. Surface morphologies and crystal structures were investigated through scanning electron microscopy and Raman spectroscopy. Furthermore, different batches of sensors have been prepared, and their sensing performances towards carbon monoxide and nitrogen dioxide have been explored. Moreover, metal oxide nanowires have been integrated into an electronic nose and successfully applied to discriminate between drinking and contaminated water.

## Introduction

Nanotechnology is the base for improving knowledge about materials and phenomena at the nanometric scale. This is crucial for the development of any device. Because sensors are able to acquire chemical information from the surroundings in real time, they are attracting a lot of interest and, therefore, they are going to have an increasing impact on everyday life. Chemical sensors may detect toxic analytes and explosives, be integrated in security systems to protect workers from chemical hazards, be used for environmental or health and wealth monitoring, and for food-chain control. In the last years, much effort has been taken to increase the quality and security of the food chain, since the ingestion of food not properly stored or treated is one of the most frequent reason of hospitalization [1].

Chemical sensors may play a pivotal role in all these applications. Metal oxides were the first to be commercialized as conductometric chemical sensors in form of thick films, and they are still the most promising materials for chemical sensing [2–3]. Metal oxide chemical sensors are more stable and reproducible compared to organic sensors. However, in order to be used in real cases, these devices need to meet many requirements such as high sensing performances in terms of sensitivity, selectivity, response kinetics and reliability [4]. The design of active materials is essential and it must be the starting point for the control of the functional parameters of the final device. Nonetheless, great attention must be paid to the integration of the active material onto the transducer. In order to have a stable chemical sensor, not only the active material, but all the components of the device, such as electrical contacts and heating system, must be stable and reliable.

The scope of this manuscript is to present different techniques for the preparation of nanostructures, and to show the different sensing capabilities of oxides within a real application, using sensors arrays and electronic noses. Evaporation, thermal oxidation and hydrothermal methods were optimized for the direct integration of metal oxide nanowires into chemical sensor transducers, without using any transfer method that may not guarantee the stability and reproducibility required for potential commercial devices. The deposition has been directly performed on the functional substrates, avoiding post-processing transfer techniques that may decrease the adhesion and therefore the mechanical and electrical stability of the final devices.

In order to prepare an array of sensors, different metal oxides have been studied, both conventional and new ones. We have investigated different preparation techniques and materials and we have compared their sensing properties towards two well-known and studied species (an oxidizing and a reducing gas interesting for environmental monitoring). Moreover, we have integrated metal oxide nanowires into an electronic nose and proved its ability in a real case study, more specifically the detection of water contamination.

## Results and Discussion

### Preparation of metal oxide nanostructures

#### Evaporation–condensation technique: NiO, SnO_2_ and ZnO

**Figure 1:**
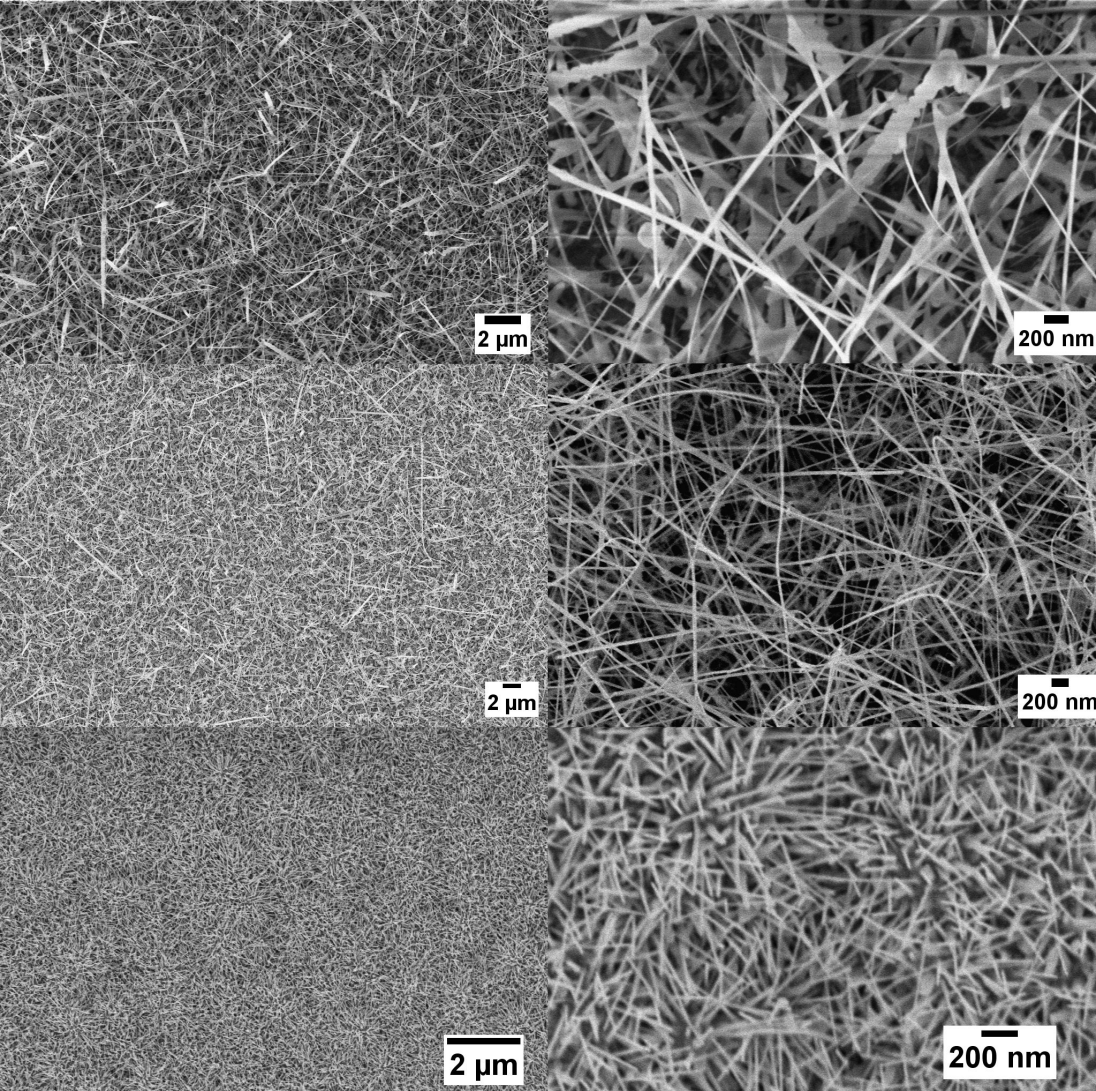
FE-SEM images of NiO nanowires at different magnifications (top), SnO_2_ nanowires (middle) and ZnO nanowires (bottom).

Evaporation–condensation allows one to obtain disordered mats of nanowires, covering the area of substrates with the catalyst. Figure 1 (top) shows the FE-SEM images of NiO nanowires at different magnifications, while Figure 1 (middle) and Figure 1 (bottom) report SnO_2_ nanowires and ZnO nanowires, respectively. Nanowires were directly grown on the active substrates used for functional characterization. It has been observed that the NiO nanowires were grown thin and long and they showed a dense morphology covering the whole substrate. The diameters of these nanowires were found to lie in the range of 20 to 60 nm. The same holds for tin oxide nanowires, even if in this case nanowires are distributed more uniformly on the substrates. ZnO nanowires exhibit a smaller average diameter (20–50 nm), but they are also shorter and form a very dense mat.

**Figure 2:**
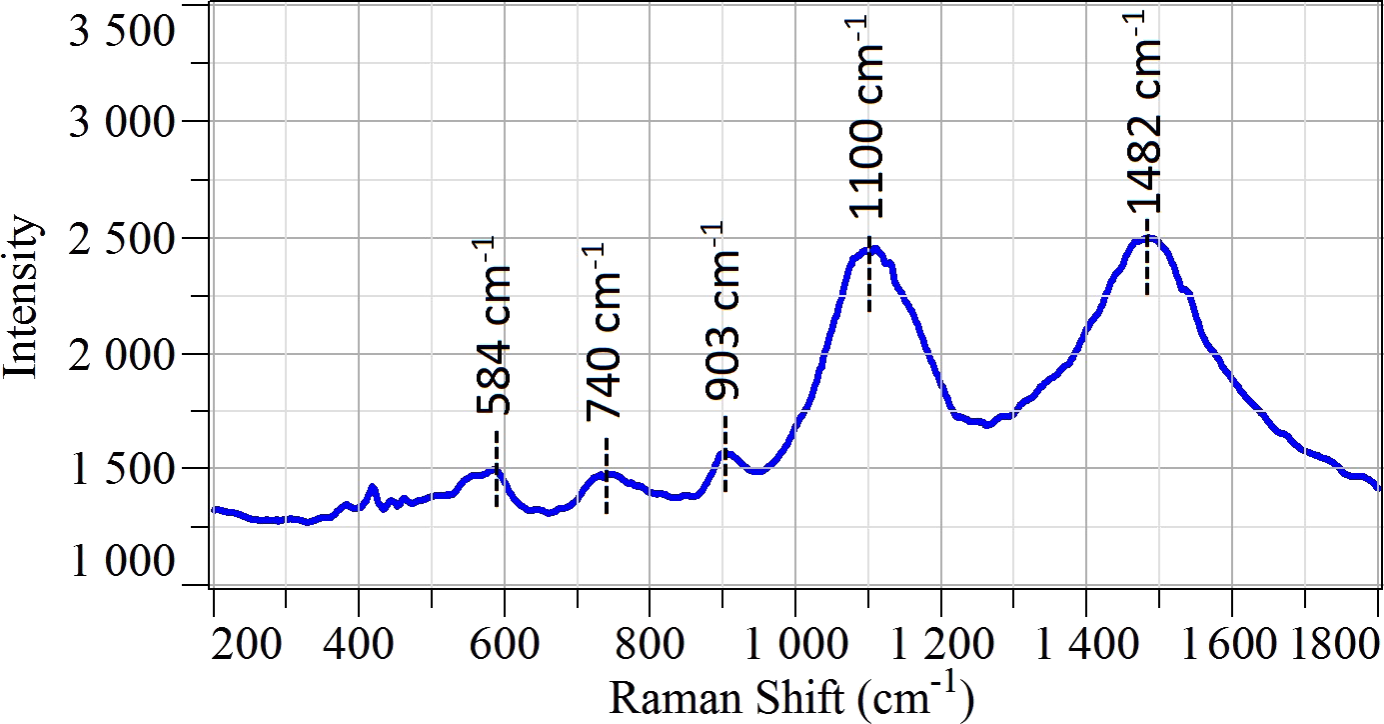
Raman spectrum of NiO nanowires deposited on alumina substrate measured in ambient air at room temperature.

Raman spectrum of NiO nanowires (Figure 2) shows several bands above 400 cm^−1^. The first four bands at 584 cm^−1^, 740 cm^−1^, 903 cm^−1^ and 1100 cm^−1^ have vibrational origin. The first band at 584 cm^−1^ corresponds to one phonon TO (transverse mode) and LO (longitudinal mode), 740 cm^−1^ to the two phonon 2TO modes, 903 cm^−1^ to the TO+LO and 1100 cm^−1^ is related to the 2LO modes. One last strong band was observed at 1482 cm^−1^ and it belongs to magnon (2M) scattering [5]. The extra peak that appears in the spectra below 450 cm^−1^ belongs to the alumina substrate.

**Figure 3:**
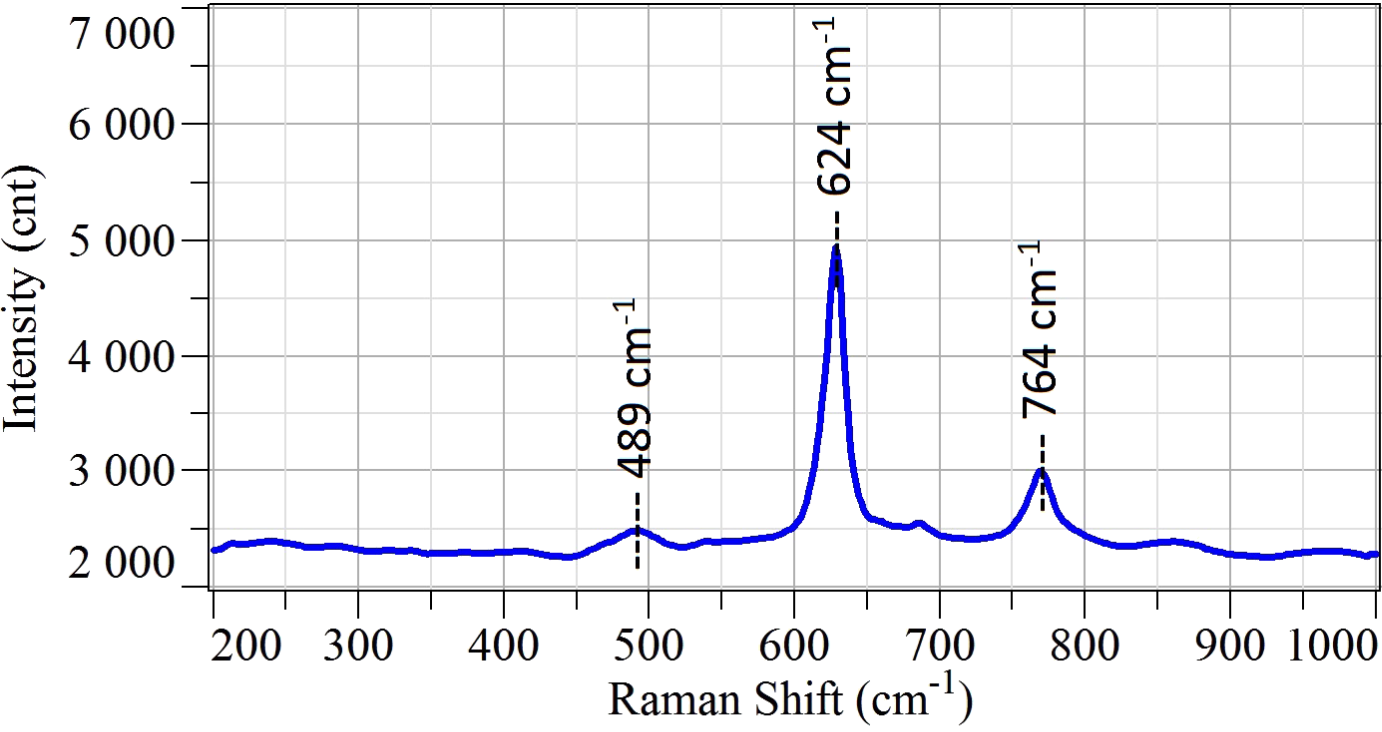
Raman spectrum of SnO_2_ nanowires deposited on alumina substrate measured in ambient air at room temperature.

Concerning SnO_2_, three major peaks are detected in the Raman spectrum in Figure 3. The peaks located at 489, 624 and 764 cm^−1^ are related to E_g_, A_1g_ and B_2g_ vibration modes, respectively. These peaks are the common Raman peaks of tetragonal rutile bulk SnO_2_, as reported in literature [6].

**Figure 4:**
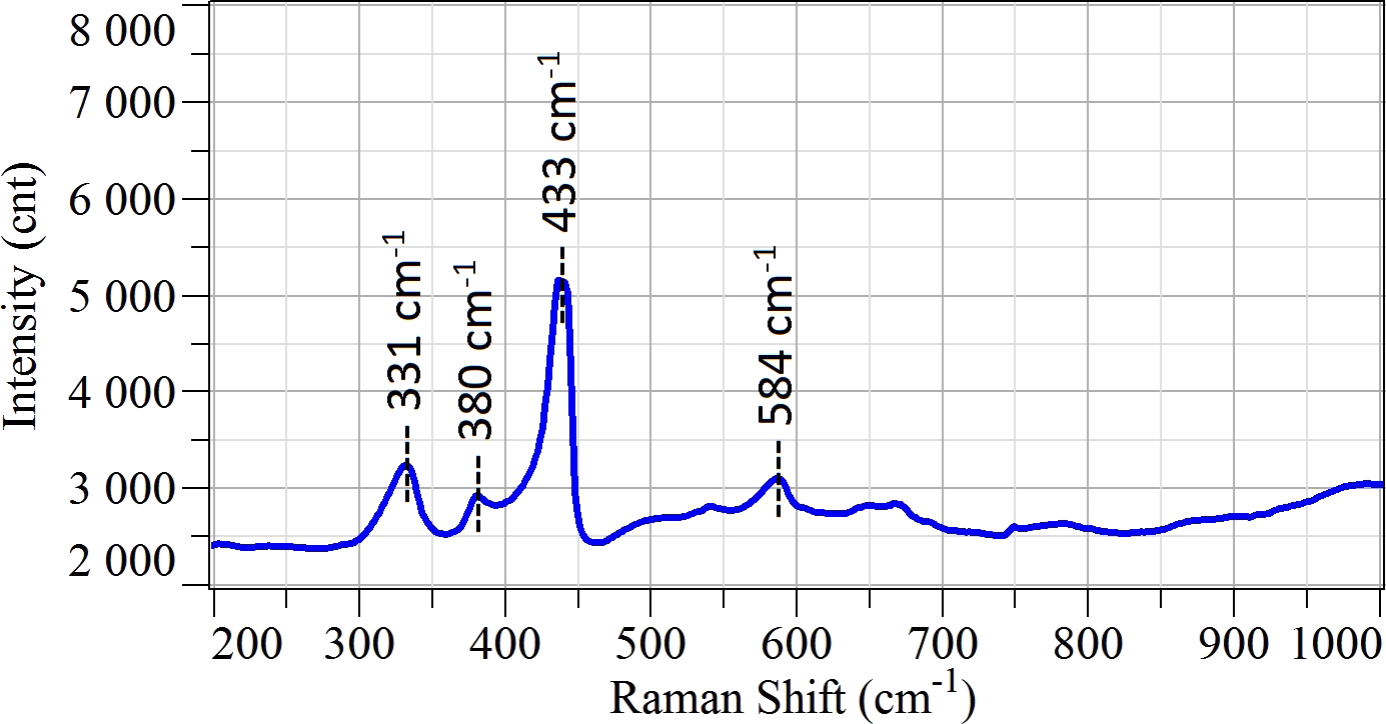
Raman spectrum of ZnO nanowires deposited on alumina substrates measured in ambient air at room temperature.

The ZnO Raman spectrum is reported in Figure 4. Typical modes for ZnO crystals are a longitudinal optical (LO) mode, measured at 584 cm^−1^, and the transverse A_1_ mode, measured at 380 cm^−1^. Moreover, there are one E_2_ vibration at 433 cm^−1^ and one transverse (TO) mode E_1_ at about 400 cm^−1^, which is contributing to the tail of the E_2_ peak. The signal at 331 cm^−1^ is a second-order vibration.

#### Thermal oxidation technique: WO_3_

**Figure 5:**
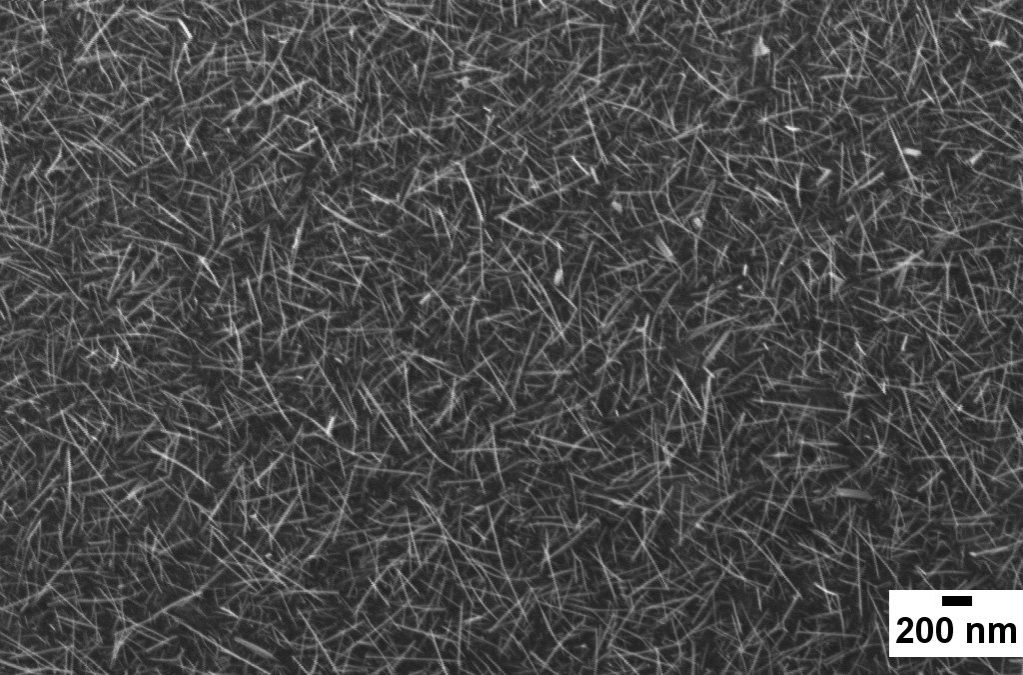
SEM picture of WO_3_ nanowires on alumina substrate.

Thermal oxidation of metallic tungsten films resulted in a disordered mats of tungsten oxide nanowires, covering all the patterned area of the substrates. Figure 5 reports a SEM picture of the nanowires, at 50k magnification, highlighting the lack of a preferred orientation on the substrate. The average diameter of the nanowires is very small (20–30 nm) while the length is approximately 1–2 μm.

**Figure 6:**
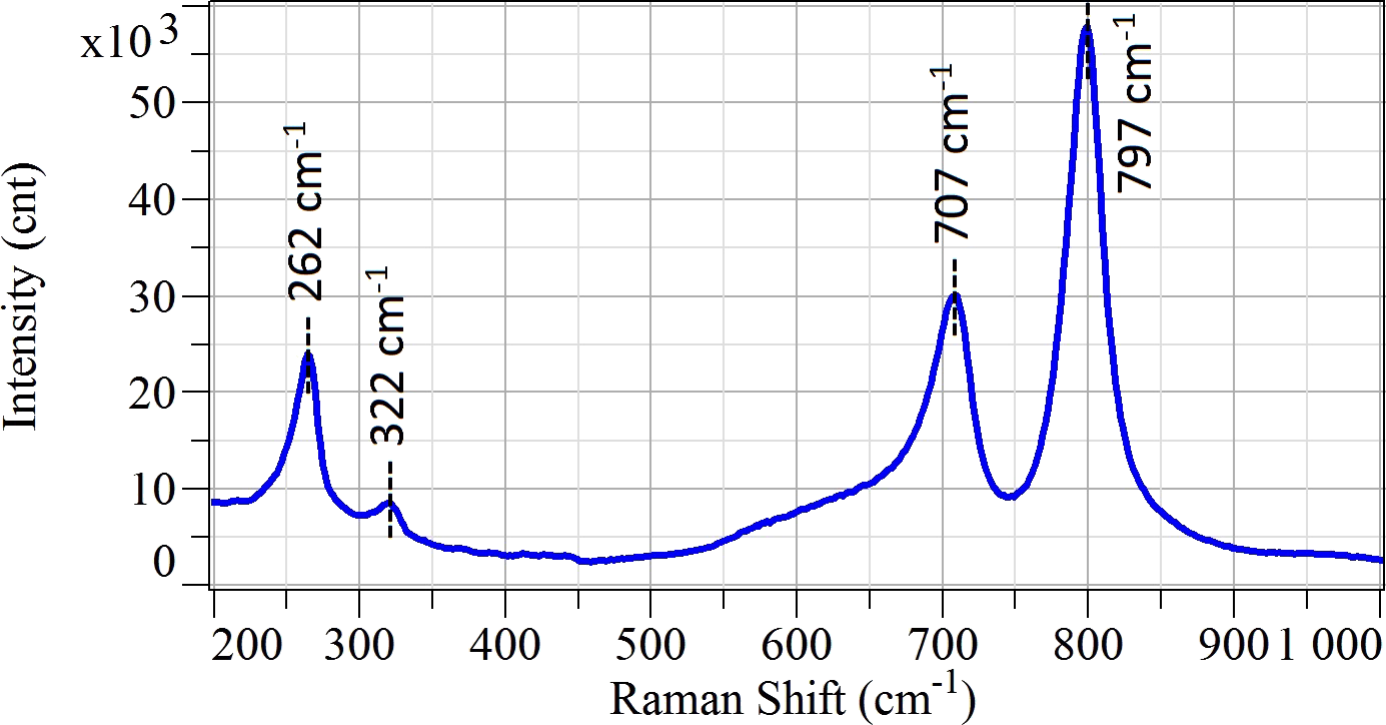
Raman spectrum performed on WO_3_ nanowire deposited on alumina substrate measured in ambient air at room temperature.

Raman spectroscopy was performed on the samples to evaluate the crystallinity and the composition of the material. Figure 6 reports the Raman spectrum of WO_3_ nanowire networks. All identified peaks can be attributed to tungsten trioxide, while there is no sign of alumina (corundum) peaks related to the polycrystalline substrate. This means that tungsten oxide covers the entire substrates. More specifically, the peaks at 707 cm^−1^ and 797cm^−1^ are related to the stretching vibration of bridging oxygen in (W–O–W) bonds [7], and they are typical peaks of monoclinic tungsten trioxide. The other two major peaks at 262 cm^−1^ and 322 cm^−1^ are due to the bending vibration of (O–W–O) bonds [8].

#### Hydrothermal technique: Nb_2_O_5_

**Figure 7:**
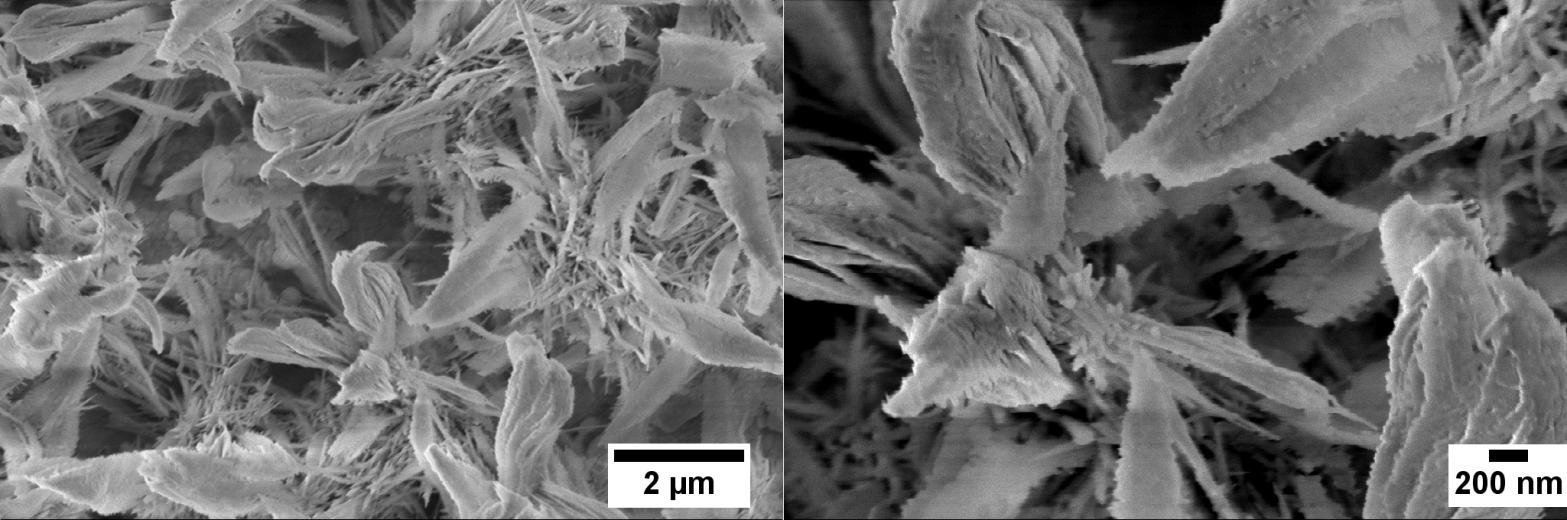
SEM images of Nb_2_O_5_ nanoflowers at 25k (left) and 75k (right) magnification level.

During the preparation of niobium oxide nanostructures using a hydrothermal technique, during the first experiments we had adhesion problems of the metal layer to the substrates. We changed the thickness of the film, working temperature, KOH molarity and time in order to find out the best conditions to synthetize niobium oxide nanostructures directly onto the substrate. Once we achieved reproducible results, nanostructures were investigated in terms of morphology, structural features and functional properties. By using scanning electron microscopy (SEM, LEO 1525) it was possible to verify the presence of the nanostructures on alumina substrates and show their morphology. During the hydrothermal treatment Nb_2_O_5_ nanoflowers were formed (Figure 7).

**Figure 8:**
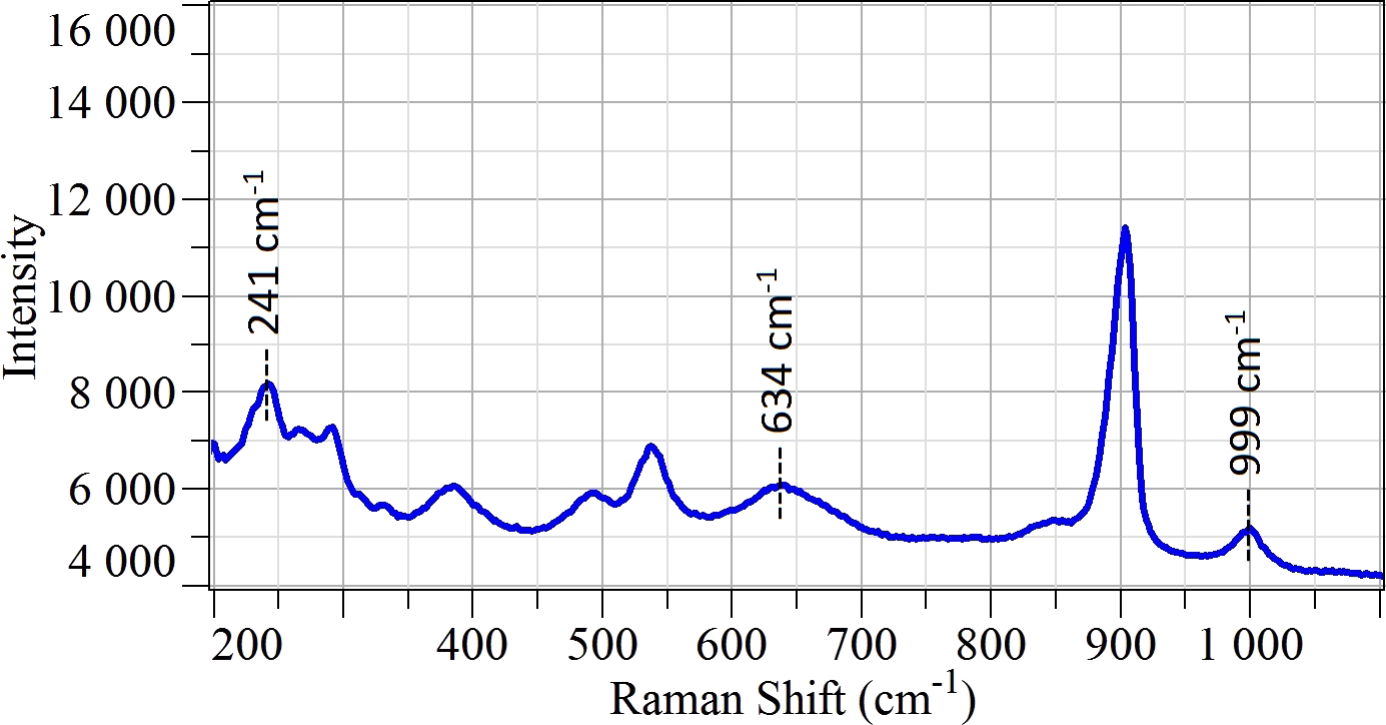
Raman spectrum of Nb_2_O_5_ nanoflowers deposited on alumina substrate measured in ambient air at room temperature.

Raman spectroscopy was performed in order to scrutinize the structural properties of the material. The analysis showed the presence of niobium oxide with residual potassium from the use of KOH during the hydrothermal treatment (Figure 8).

The niobium(V) oxide peaks in Figure 8 (marked by dashed lines) match the ones reported in literature [9–10]. The peak at 999 cm^−1^ corresponds to longitudinal optical mode (LO) of Nb–O stretching associated to NbO_6_ octahedra. The corresponding transverse optical (TO) mode is observed at 634 cm^−1^. The band close to 241 cm^−1^ is related to Nb–O–Nb bending [10]. The remaining peaks are related to residual potassium, which forms a K*_x_*Nb*_y_*O*_z_* ternary compound, specifically K_4_Nb_6_O_17_ [11]. Due to the growth technique used, it is very difficult to separate the contribution of pure Nb_2_O_5_ from K_4_Nb_6_O_17_. In literature, there are no reports on the sensing performance of this ternary compound as chemical sensor. However, it is a promising material for water splitting applications [12–13].

### Functional tests

After morphological and structural analysis, we investigated the functional properties of these active materials as chemical sensors. In general, when a metal oxide such as SnO_2_ is exposed to dry air [14–15], there is adsorption of oxygen molecules on the surface, leading to the formation of active oxygen species such as O^−^, O^2−^ and O_2_^−^. These adsorbed ions trap free electrons from the surface, thus changing the overall electrical conductance of the material. When we release a reducing gas species in the atmosphere, gas molecules interact with these pre-adsorbed oxygen ions releasing electrons on the surface of the material. This injection of free carriers results in an increase (for n-type semiconductors) or decrease (for p-type semiconductors) of the electrical conductance, respectively. For example, if we consider CO as target species to be detected we have (Equation 1) [14]:

[1]
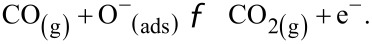


In the presence of oxidizing species such as NO_2_, the interaction on the oxide surface leads to an increase of the number of adsorbed species from the gas phase. This results in an increase of electrical conductance for for p-type materials and, correspondingly, a decrease for n-type materials. This behaviour can be better explained through the following reactions in dry air [15]:

[2]
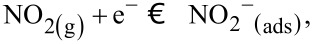


[3]
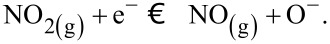


The role of water vapour also needs to be taken into account [16–18], but its effect on the sensing mechanism strongly depends on the used material. For example, it has been demonstrated that for SnO_2_, humidity competes with reducing gases for the same reactive oxygen species, and it has a site blocking effect when interacting with the surface [16–17]. For this reason, in presence of humidity the concentration of adsorbed oxygen is lower, and the response of the SnO_2_ sensor to CO is lower than under dry conditions. On the contrary, the lattice of WO_3_ is oxidized by humidity, and more reaction sites for CO are present on the surface than in dry air [18].

**Figure 9:**
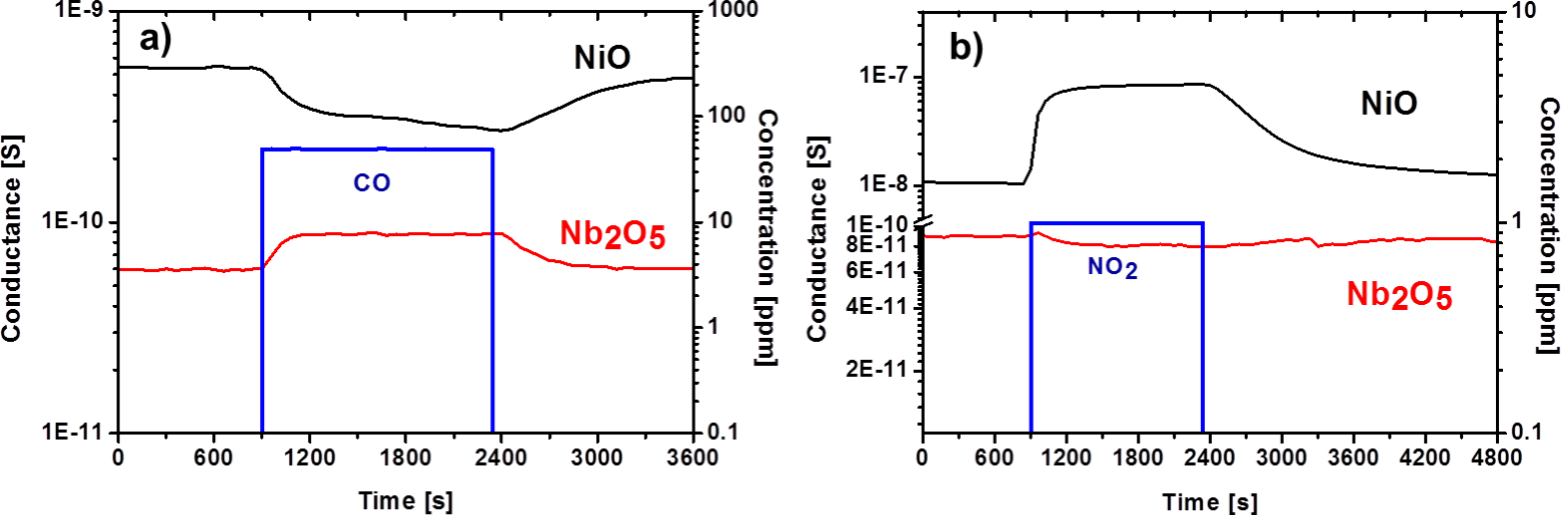
Dynamic response of NiO and Nb_2_O_5_ sensing devices towards (a) (CO; 50 ppm, NiO (300 °C) and Nb_2_O_5_ (400 °C)) and (b) (NO_2_; 1 ppm, NiO (200 °C) and Nb_2_O_5_ (400 °C)) measured at a relative humidity of 50% at 20 °C.

As an example, we report in Figure 9 the dynamic response of NiO (p-type) and Nb_2_O_5_ (n-type) devices. In the presence of reducing CO we observed a decrease in electrical conductance for NiO sensor and an increase in case of Nb_2_O_5_.

Theoretical calculations have shown that the response of p-type metal oxide semiconductors toward a specific target compound should be equal to the square root of that of an n-type metal oxide semiconductor under the same conditions (morphology, structure). This is due to the intrinsic nature of the charge carriers of p-type materials (holes) compared to those of n-type materials (electrons) [19–20]. However, this does not take into account the specific catalytic properties of each material. For some applications p-type materials could perform better than n-type materials.

Moreover, the response of a metal oxide gas sensor is influenced also by the morphology, and specifically by the size of the nanostructures [21]. In this work, we investigate the preparation of low-dimensional metal oxide nanowires. However, because of the growth process used and the nature of the materials themselves, it is difficult to have exactly the same nanostructures for all five different materials and, therefore, a direct comparison is difficult. It is a very complex task to desribe the sensing mechanism of each semiconducting metal oxide. It requires a comprehensive characterization of the materials together with a deep understanding of the surface reactions. Operando measurements, in which electrical measurements and spectroscopic investigations are performed simultaneously on the material, have proven to be a powerful investigation tool in answering some questions about the sensing mechanism [22–23].

**Figure 10:**
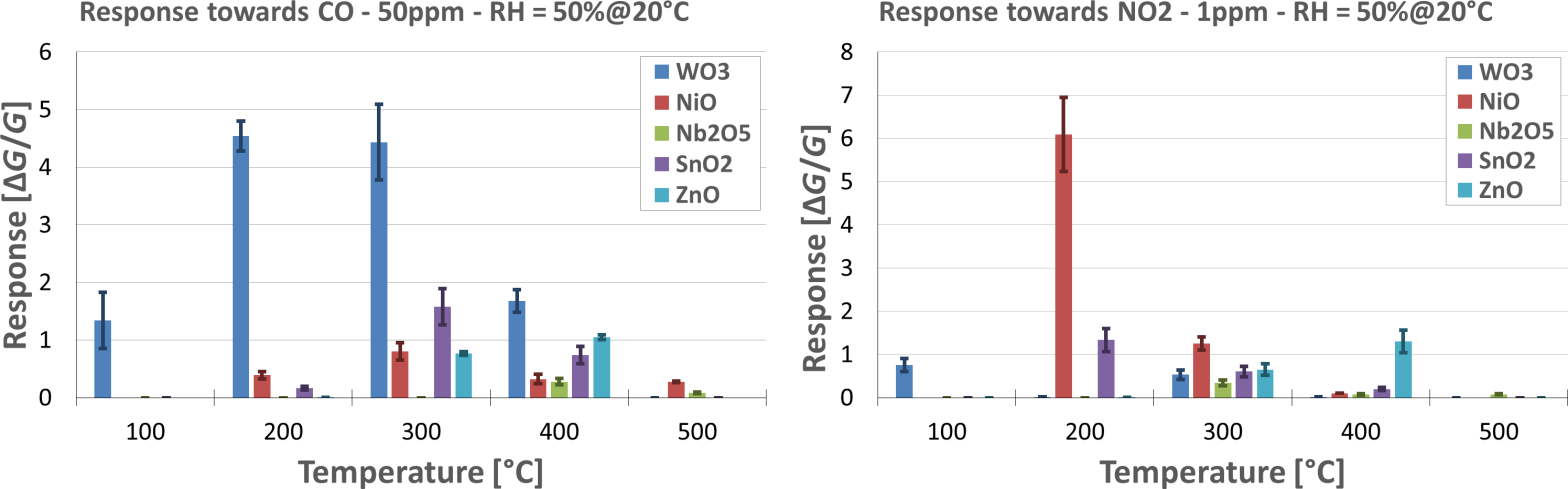
Sensor response towards 50 ppm of CO (left) and 1 ppm of NO_2_ (right) as a function of the temperature. The relative humidity was kept constant at 50%, the temperature was 20 °C.

In the present manuscript, we report the sensing performance of five different nanostructured metal oxides, synthetized directly on the transducers used to fabricate the final device. A temperature screening was performed in order to identify the optimal working temperature of each material in the detection of the two target chemical compounds. Results are reported in Figure 10. As expected, each material behaves differently, and the working temperature has a strong effect on the response. Although some materials are more suited than others to detect CO or NO_2_, it is important to mention that all metal oxides exhibit cross-sensitivity to other chemical species too. This lack of selectivity toward specific chemical species is one of the major drawbacks of the conductometric use of metal oxides. However, an array of devices based on different materials, each with its own sensing properties (a so-called electronic nose), could provide a robust and versatile tool for the unambiguous detection of volatile compounds.

Considering carbon monoxide as target gas, it is evident that WO_3_ is the most sensitive material, at almost every temperature. The optimal temperature is 200 °C, exhibiting a response of about 4.5 to 50 ppm of CO. The response of other materials is significantly lower, and the optimal working temperature is higher than that of WO_3_. The excellent performance of WO_3_-based devices could be related to the very small dimensions of the nanowires. However, the sensing mechanism of WO_3_ toward CO may differ from the one of other metal oxides. In particular, former studies [23] suggest that CO can locally reduce the surface of WO_3_ nanowires, strongly influencing the electrical conductance of the surface. This is not observed, for example, in sensors based on SnO_2_ nanowires. Unfortunately, up to now we do not have enough information to determine which parameter has the biggest impact on the sensor response.

Concerning nitrogen dioxide instead, NiO is very sensitive, more than all other materials. The optimal working temperature of NiO is 200 °C, with a response of about 6 to 1 ppm of NO_2_. At lower temperatures (100 °C), NiO devices are too resistive to be measured in our test chamber. NiO has hardly been studied as a material for chemical sensors. Hence, there are only few reports about a tentative NO_2_ sensing mechanism. Zhang et. al. [24] pointed out that nickel vacancies could play an important role in the interaction between NO_2_ and the NiO surface and in the sensing mechanism in general.

**Figure 11:**
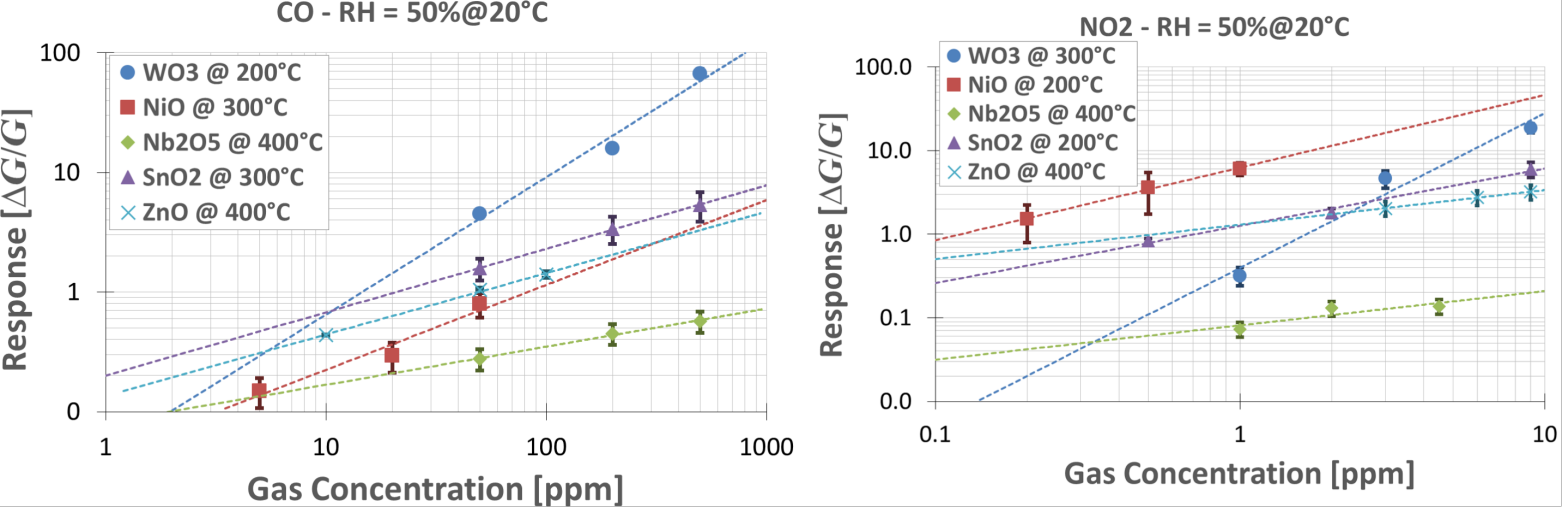
Calibration curves and power-law fitting for CO (left) and NO_2_ (right). The relative humidity was kept constant at 50%, the temperature was 20 °C.

The calibration curves reported in Figure 11 exhibit results similar to those of the temperature screening. WO_3_ is the best-performing material for the detection of CO, at concentrations higher than 10 ppm. At lower concentrations, tin-oxide-based devices exhibit a higher response, according to the preliminary calibration curve estimated from the measurement by power-law fitting. If the devices are used for the evaluation of air quality in closed spaces [25], it may be necessary to detect concentrations of carbon monoxide lower than 10 ppm. Therefore tin-oxide-based devices are ideal candidates.

On the contrary, NiO performs much better than other oxides for NO_2_ detection, especially at low concentrations (below 10 ppm). European Union (EU) Air quality Standards require the average concentration of NO_2_, in a period of one hour, to be lower than 0.1 ppm [25]. The response of NiO-based devices is sufficiently high to easily detect such concentrations, exhibiting a response of one at 0.1 ppm. At higher concentrations (above 10 ppm), WO_3_ could be a better choice for the detection of nitrogen dioxide, according to the raw estimation from the power-law fitting. However, such high concentrations are not interesting for environmental monitoring, being far above the air quality requirements [25].

Considering both target gases, Nb_2_O_5_-based sensors exhibit the lowest performance. One explanation could be the higher dimensionality (3D) of these nanostructures compared to nanowires (1D), originating from the aggregation of 1D or 2D objects (such as nanowires, nanorods, nanosheets) occurring during the growth [26]. In particular, nanowires exhibit a very high surface-to-volume ratio, and have a large surface area, while the performance of the Nb_2_O_5_ nanoflowers depends on the size of the nanostructures and how they aggregate to the 3D structure. If the assembly leads a low surface-to-volume ratio, the final performance could be degraded.

Reproducibility was also taken into account. For the same material, a batch of different devices was fabricated and measured. In Figure 10 and Figure 11, the average values of these measurements are reported. The variation of the response of an individual sensor is around 5%. However, in case of sensors produced in different batches it may increase up to 20%. The stability of the devices over medium-term operation was very good. The measurements were not performed sequentially and each sensor was measured over a time of more than two months. Within this interval, sensors continued to work properly, without any significant breakout or loss of performance.

The proposed chemical sensing devices, based on nanostructured materials, were compared to current state-of-the-art devices. Literature data of the performance toward CO and NO_2_ of some similar nanostructures were collected in Table 1. Unfortunately, it was not always possible to find similar gas concentrations, especially for Nb_2_O_5_. This material has hardly been studied in chemical sensors. Therefore, there are not many reports on its sensing performance. Comparing the most responsive materials to CO and NO_2_ (WO_3_ and NiO, respectively) with the current state of the art, the presented nanostructures outperform the results reported in literature with similar morphologies.

**Table 1:** Sensing performance reported in literature for some similar metal oxide nanostructures toward CO and NO_2_.

structure	method	gas/concentration	response	transfer of nanostructures	ref.

nickel oxide					

NiO thin film	sol–gel and spin coating	NO_2_/200 ppm	0.233@200 °C	no	[27]
NiO nanosheets	hydrothermal method	NO_2_/20 ppm	0.8%@250 °C	yes	[28]
NiO thin film	DC reactive magnetron sputtering	NO_2_/5 ppm	2.6@160 °C	no	[29]
NiO nanostructures	hydrothermal reflux process	CO/100 ppm	1.14@100 °C	yes	[30]
NiO thin film	DC reactive magnetron sputtering	CO/200 ppm	0.3@420 °C	no	[31]

tungsten oxide					

WO_3_ porous thin film	DC magnetron sputtering and anodic oxidation	NO_2_/1 ppm NO_2_/5 ppm	40@150 °C ca. 100 @150 °C	no	[32]
WO_3_ nanowires	vapor transport method	NO_2_/5 ppm CO/200 ppm	7.8@200 °C 1.4@200 °C	yes	[33]
WO_3_ nanowires	thermal evaporation	NO_2_/10 ppm CO/100 ppm	146@250 °C 1.3@250 °C	no	[34]
WO_3_ nanorods	thermal evaporation	CO/30 ppm	0.023@300 °C (bare WO_3_) 0.0482@300 °C (Pt–WO_3_)	yes	[35]

zinc oxide					

ZnO nanocrystalline thin film	spray pyrolysis	NO_2_/7 ppm	3.32@200 °C	yes	[36]
ZnO nanopyramids	non-aqueous route	NO_2_/10 ppm	14.5@200 °C	yes	[37]
ZnO cacti-like structures (Zcc) & nanoneedles (Znn)	chemical route	NO_2_/200 ppm	0.89@200 °C (Zcc) 0.64@200 °C (Znn)	no	[38]
ZnO nanocrystalline nanowires	thermal evaporation	CO/1000 ppm	51.64@300 °C	yes	[39]
ZnO nanorod array	hydrothermal route	CO/200 ppm	2.2@250 °C	no	[40]

tin oxide					

SnO_2_ thin film	sol–gel and spin coating	NO_2_/100 ppm	0.019@200 °C	yes	[41]
SnO_2_ nanoribbons	thermal deposition process	NO_2_/3 ppm	1.16@rt	yes	[42]
SnO_2_ hollow spheres	solution phase deposition	NO_2_/5 ppm NO_2_/20 ppm NO_2_/50 ppm NO_2_/100 ppm	1150@160 °C 2031@160 °C 2471@160 °C 2229@160 °C	yes	[43]
SnO_2_ nanowires	thermal evaporation	CO/100 ppm	2.9@400 °C	no	[44]
SnO_2_ thin film	hydrothermally treated sol solution	CO/800 ppm	489.6@350 °C 52.4@200 °C	yes	[45]

niobium oxide					

Nb_2_O_5_ nanowires	thermal oxidation process	CO/200 ppm	ca. 0.1@rt	no	[46]
Nb_2_O_5_ thin film	RF sputtering	CO/50 ppm	ca. 1.66@350 °C	yes	[47]
monodispersed Nb_2_O_5_ microspheres	solvothermal route	NO_2_/5 ppm	ca. 2@450 °C	yes	[48]

### “Small sensor system” tests

In order to study these materials in real applications, metal oxide nanowires have been integrated in an electronic nose called “Small Sensor System” (S3). Nanowire devices were integrated together with conventional thin film counterparts, to see if the discerning ability of the electronic nose is affected by the integration of nanostructured active materials. In particular, we decided to integrate tin dioxide and zinc oxide devices, since these are the most widely used and studied materials for chemical sensors.

**Figure 12:**
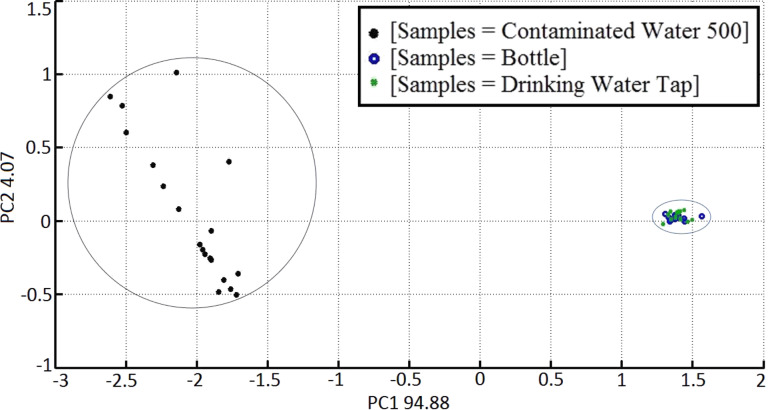
Principal component analysis (PCA) score plot for drinking water (blue and green dots) and a solution with 500 CFU/mL pathogenic microorganisms (black dots).

As a case study, we have chosen the analysis of water contamination. Nowadays, a fast and economic device for the early detection of microbial contamination and quality assurance is needed to reduce the number of food-borne related hospitalizations by year. Moreover, there is a strong need for a portable user-friendly device with low power consumption. Our analysis was performed to verify whether S3 is able to distinguish between drinking and contaminated water. The concentration of pathogenic microorganisms in the contaminated sample is 500 CFU/mL (CFU = colony-forming units). Pathogenic microorganisms are always used as an indicator of the water quality. In particular, *E. coli* normally appears at concentrations of 100 to 2 × 10^4^ CFU/100 mL [49]. In this study, we analysed contaminated water samples in the first 24 h after sampling. For each contaminated and uncontaminated sample, 40 analyses were performed, for a total of 80 measures. This was necessary to get a substantial statistical ensemble. The principal component analysis (PCA) score plot graph in Figure 12 reports the data corresponding to contaminated and uncontaminated water. It is evident that the measurements of these two samples are well separated into two different clusters. Therefore, the analysis is able to distinguish between contaminated water and drinking water.

SnO_2_ nanowires S3 sensors perform best in terms of response, compared to their thin film counterpart for the class of VOCs analysed. The S3 was able to achieve information about the whole fingerprint emitted from contaminated and uncontaminated water. At the same time the S3 device is not able to identify, without training, a specific VOC. In order to gain more information about the VOCs, analysis with classical chemical techniques such as GC-MS-SPME was carried out simultaneously revealing the most representative compounds related to microbial grow (Table 2).

**Table 2:** Volatile chemical compounds identified, using SPME-GC-MS, in the contaminated water.

retention time [min]	compound

1.646	2-methylbutene
1.726	2-methyl-1,3-butadiene
2.271	4-methyl-2-hexanone
2.436	*trans*-3,5-methoxycyclohexanone
2.453	acetone
3.129	5-methyl-2-phenyl-1*H*-indole
6.208	2-fluoropropylene
10.316	5-methyl-2-hexanone
10.844	1,8-cineol
10.969	pentanol
10.975	3-methyl-1-butanol
11.759	2,4-nonadienal
13.304	2-octanone
14.919	4-methylpentane
14.923	formic acid methyl ester
16.491	acid 5-alpha-2-cholestanol
16.781	heptanol
17.111	2,5-dimethylfuran
17.440	1-octadecylamine
17.991	benzaldehyde
18.076	hexadecanol
18.230	octanol
18.690	dimethyl methylphosphonate
19.042	2-(2-methoxy)butanol
19.105	octane
19.292	4-methylthiazole
19.298	caproic acid

**Figure 13:**
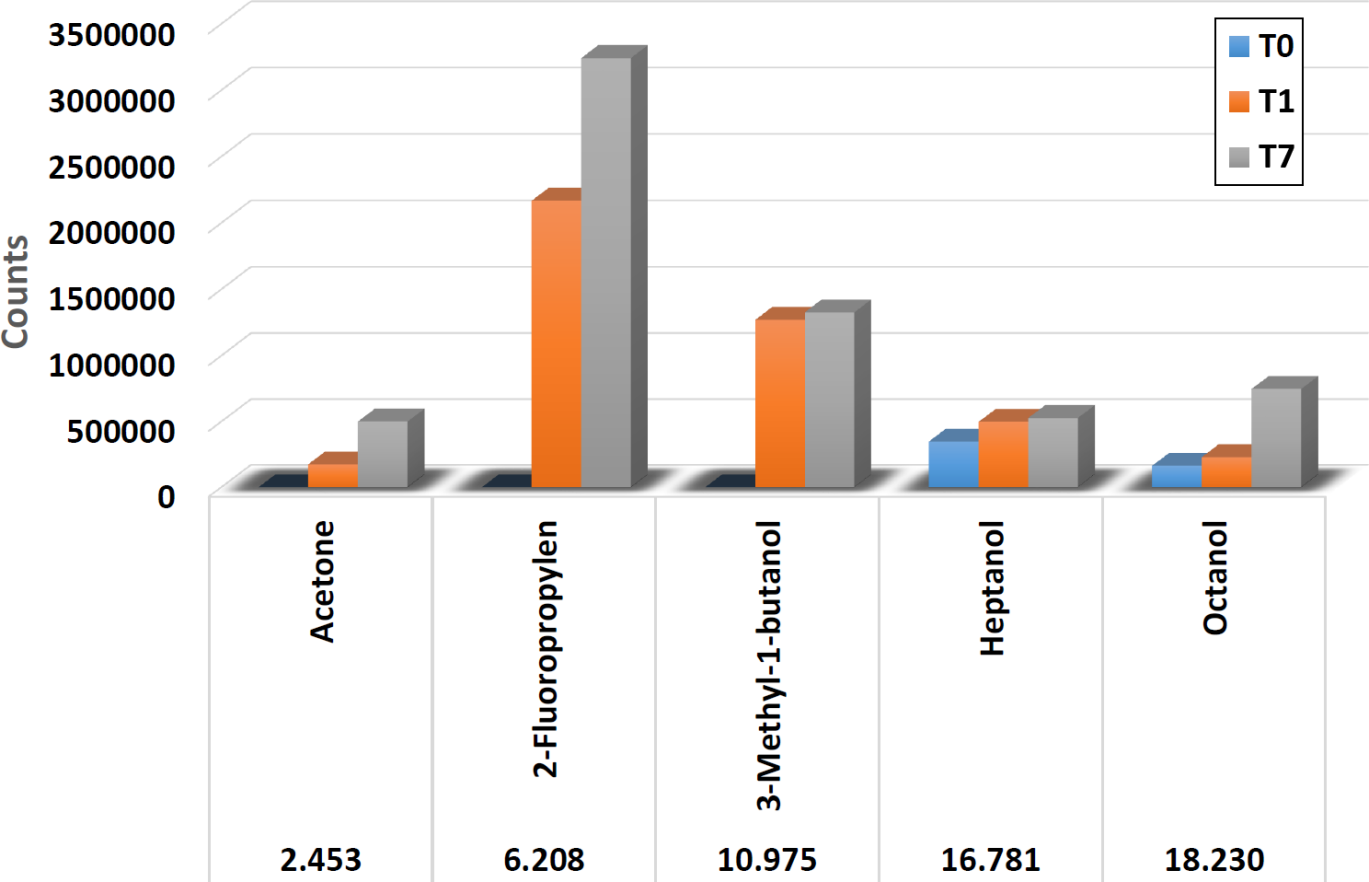
Content of VOCs over seven days of analysis.

In Figure 13, the content of five volatile organic compounds (VOCs) in the sample with contaminated water is given over a period of seven days. T0 is the day of inoculation, T1 is the measurement 1 day after inoculation (24 hours) and T7 is the measurement 7 days after the inoculation. Acetone, 2-fluoropropylene and 3-methyl-1-butanol are neoformation VOCs, because they were not present at T0. These compounds formed during microbial growth. Heptanol and octanol were present at T0 and their concentration increased. There is a defined and individual growth mode for every microorganism correlated with optimal time and temperature, and every step of the microbial growth is associated with a specific VOC.

## Conclusion

We have successfully obtained nanostructures, in form of wires or flowers, of the oxides of nickel, tungsten, niobium, zinc and tin. The structures were directly deposited on functional substrates using various techniques and procedures. We have demonstrated that a direct integration with all the three presented deposition techniques is possible, which is an essential feature to commercialize chemical sensors based on these structures.

Surface morphology and composition were studied through scanning electron microscopy and Raman spectroscopy, respectively, confirming the high surface-to-volume ratio (fundamental for chemical sensing). Moreover, we tested the functional properties with respect to chemical sensing. Different batches of sensors have been prepared, and their sensing performances towards carbon monoxide and nitrogen dioxide have been explored. The results show that the best-performing materials for detecting CO are WO_3_ and SnO_2_, while NiO performs much better than other materials in the detection of NO_2_, especially at low concentrations.

To have a clearer concerning realistic applications not only the entire sensing system (the active material plus sensing transducer) but also the entire device integrated into an electronic nose were studied. We have carried out “real-life” tests that confirm the feasibility and clearly demonstrate the potentiality of metal oxide chemical sensors in discriminating among drinking and contaminated water.

## Experimental

### Preparation of metal oxide nanostructures

Alumina substrates (2 × 2 mm^2^, 99% purity, Kyocera, Japan) were cleaned in acetone using an ultrasonic cleaner for 15 min to remove dust and impurities from the substrates. Substrates were dried with pure compressed air. The following techniques were used to grow different metal oxide nanostructures.

#### Evaporation–condensation technique: NiO, SnO_2_, ZnO

The growth of nickel oxide (NiO), tin dioxide (SnO_2_) and zinc oxide (ZnO) nanowires was performed by evaporation–condensation on alumina substrates [50]. It consists of a controlled evaporation of metal oxide powder followed by a condensation of vapor on a catalyzing substrate. The main parameters to optimize during evaporation–condensation are the evaporation temperature of the source material and the condensation temperature at which materials start to condensate and grow as 1D nanostructure. An ultrathin layer of gold particles were deposited on alumina substrates with RF magnetron sputtering at 70 W, Ar flow 7 sccm for 5 sec, acting as a catalyst for the synthesis of nanowires.

**Figure 14:**
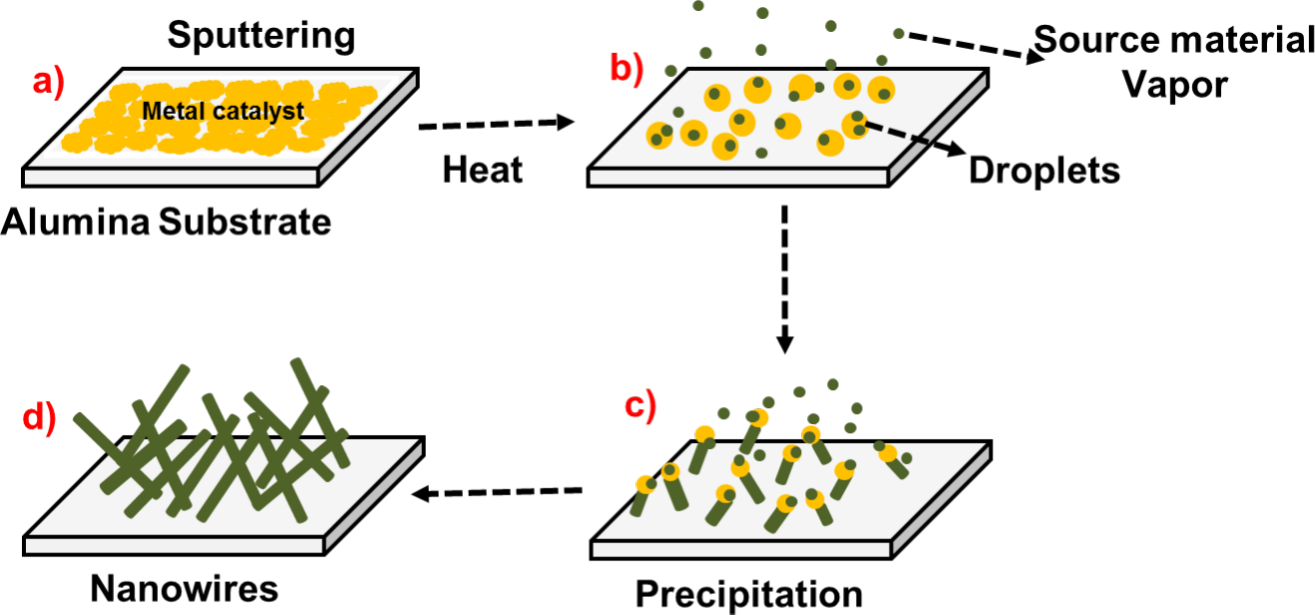
Growth of 1D structures by evaporation–condensation.

Figure 14 shows the basic mechanism of the evaporation–condensation process including three phases of material. At a certain temperature, the formation of a liquid alloy of metal and catalyst starts by absorbing vapors of the source material. As vapors of the source material are continually provided, the material starts to condensate in the form of a solid precipitate. The 1D crystal growth begins, and it continues as long as the source material is supplied [51].

The nanowire growth was carried out in a custom-made tubular furnace [52]. The evaporation temperature of NiO powder was set at 1400 °C and at 1370 °C for SnO_2_ and ZnO. Au-deposited substrates were placed at a temperature of 930 °C inside an alumina tube of the tubular furnace. Argon gas was used as a carrier gas and its flow was set at 100 sccm. Furthermore, the pressure of the tube was kept at 1 mbar for NiO and 100 mbar for ZnO and SnO_2_, with a deposition time of 15 min.

#### Thermal oxidation technique: WO_3_

Thermal oxidation is an established technique for the synthesis of copper oxide nanostructures [53]. In this work, we used this technique to synthetize tungsten trioxide (WO_3_) nanowires directly on the final transducer, starting from a metallic tungsten layer deposited by magnetron sputtering [54].

**Figure 15:**
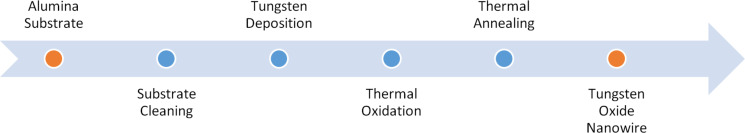
Flow chart describing the synthesis process of tungsten oxide nanowires.

Metallic tungsten was deposited by RF magnetron sputtering (100 W, 5 × 10^−3^ mbar, argon plasma, room temperature) via a shadow-mask technique, in order to obtain a 180 nm thin layer on top of the substrate. Afterwards, the samples underwent a thermal oxidation process in a tubular furnace, in reactive atmosphere. More specifically, the samples were placed in the middle of an alumina tubular furnace at a temperature of 600 °C. A dry pump was used to reach a pressure of 1 mbar inside the tube, and a flow of argon (10 mbar) was injected in the tube through a mass-flow controller (MKS, Germany). The oxidation time was 1 h. Under these conditions, the oxidation process only involves the superficial layer. After thermal oxidation, samples were thermally annealed in air for 12 h at 500 °C, to completely oxidize the material and remove all the metallic tungsten on the bottom of the nanowires. The schematic workflow to obtain WO_3_ nanowires is reported in Figure 15.

#### Hydrothermal technique: Nb_2_O_5_

In the last few years, the hydrothermal technique achieved more and more relevance in crystal growth and in particular the preparation of nanostructures because it can be used for different materials such as metal oxides, carbon nanostructures and biomaterials [55]. In this work, we applied this technique in order to obtain niobium oxide nanostructures. We started from the method explained by Fang et al. [56], but worked on a thin layer of niobium deposited on alumina substrates by magnetron sputtering. For this reason, we carried out several experiments to obtain the optimal conditions (set out below) for the growth of nanostructures. RF magnetron sputtering was used to deposit a metallic niobium film with a thickness of 500 nm on the substrates. In order to obtain this thickness, niobium was deposited for 41 min at room temperature, the power applied to the target was set to 100 W, the flow of argon was set to 7 sccm and the chamber pressure was around 5.3 × 10^−3^ mbar.

**Figure 16:**
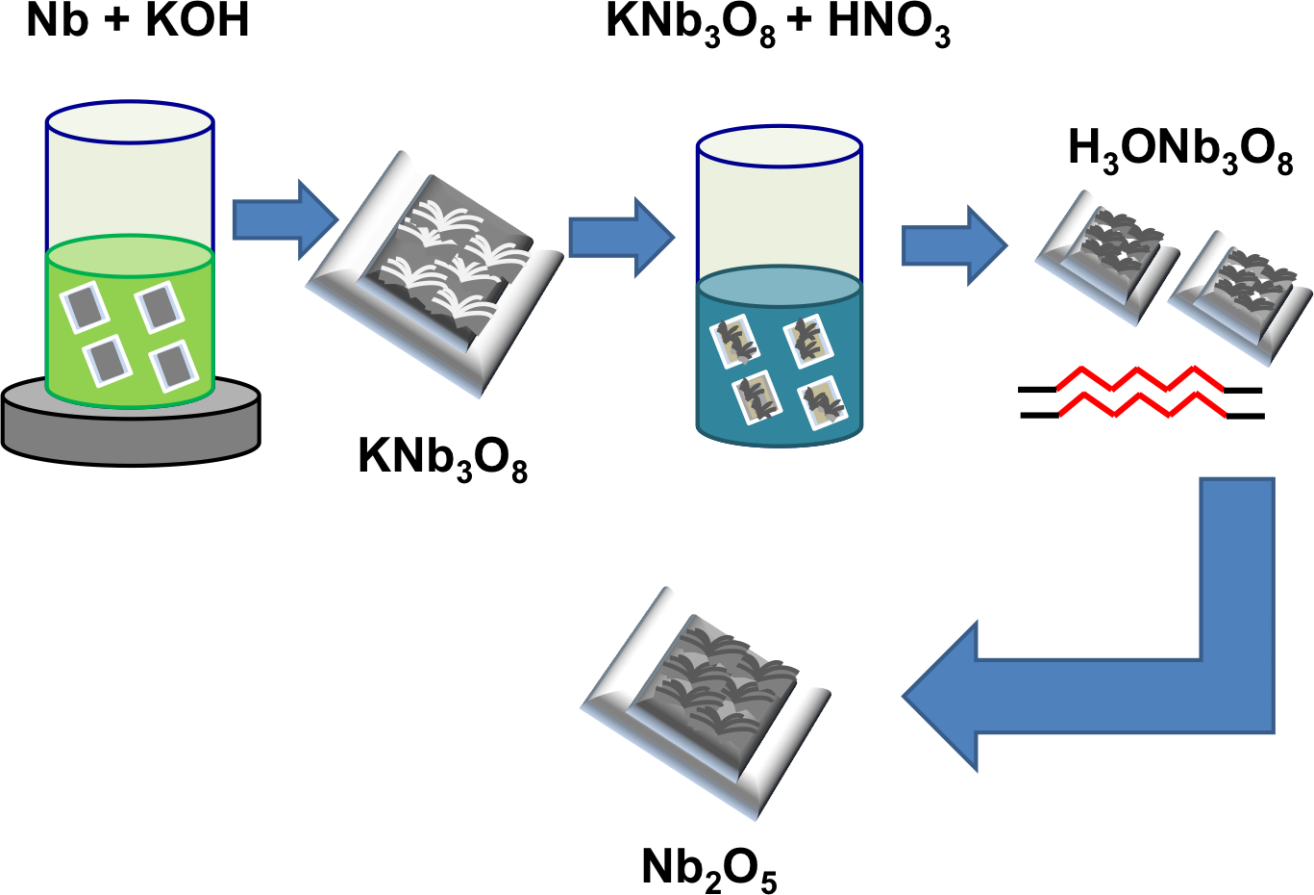
Flow chart describing the synthesis process for niobium oxide nanostructures by hydrothermal treatment.

The obtained samples were placed in a Teflon beaker with a 0.01 M KOH solution and they were placed in a high-pressure reactor. All the system was heated at 175 °C for 6 h and it was cooled down naturally. After this treatment, a white compound was present on the samples surface and an acid treatment was necessary, followed by an annealing step to obtain niobium oxide. For this reason, the prepared samples were treated with 2 M HNO_3_ solution for 48 h and then they were annealed at 650 °C for 6 h. At the end of the process Nb_2_O_5_ nanoflowers were found on the surface of alumina substrates (Figure 16).

### Characterization techniques

A field-emission scanning electron microscope (FE-SEM) LEO 1525 was used to investigate the morphology of samples. The electron beam was set at 3–5 keV energy and the samples were attached to metallic stub via carbon glue, to reduce charging effect due to the interaction of electron beam with the specimens.

Raman spectra were measured using a HORIBA monochromator iHR320, with a grating of 1800 grooves·mm^−1^ and coupled to a Peltier-cooled Synapse CCD (HORIBA). A helium–cadmium (He–Cd) blue laser (442 nm) was focused on the samples by a fiber-coupled confocal optical microscope (HORIBA) at 50× magnification. Spectra were recorded in the wavelength range of 200–1000 cm^−1^ (WO_3_, SnO_2_ and ZnO nanowires), 200–1800 cm^−1^ (NiO nanowires) and 100–1500 cm^−1^ (Nb_2_O_5_ nanostructures).

### Functional tests

Conductometric sensing devices were fabricated to integrate metal oxide nanowires in functional devices. Interdigited platinum electrodes were deposited on top of nanowires by DC magnetron sputtering (70 W, 5 × 10^−3^ mbar, argon plasma, room temperature, 1 μm thickness). On the back side of the alumina substrates, platinum heating elements were deposited using the same sputtering technique. Samples were finally mounted on TO39 packages using electro-soldered gold wires.

A flow-meter technique was used to evaluate the performance of fabricated conductometric devices for the detection of two common air contaminants, namely carbon monoxide (CO) and nitrogen dioxide (NO_2_). Samples were mounted in a test chamber of 1 L volume enclosed in a custom-made climatic chamber, set at 20 °C, to remove any influence from external ambient conditions. A fixed voltage of 1 V was applied to the sensing element of each sensor, while the current flowing was measured by picoamperemeters (Keithley). The temperature of the sensors was controlled independently by applying a known electrical power to the heaters. A temperature screening was performed, to identify the optimal working temperature of the materials. Metal oxide materials may exhibit a small drift in the electrical conductance during the heating process due to the desorption of gases, heat diffusion and mechanical stress. To reduce this effect as much as possible and thus have a stable baseline, we thermally stabilized (8 h) the samples at a selected target temperature prior to gas-sensing measurements. Different concentrations of target chemical compounds (SIAD, Italy) were injected inside the chamber for 30 min, followed by 1 h of recovery using synthetic air. The relative humidity was kept constant at 50%.

### Small sensor system

The device we used in this work is a “Small Sensor System” (S3). The sensor array is located in a thermally controlled chamber of 20 mL internal volume, where six sensors are placed: three thin films (SnO_2_–MoO_3_ [57], SnO_2_–WO_3_ [58], SnO_2_ with Ag catalyzer [59]) and three sensors based on metal-oxide nanowires (one of SnO_2_, two of ZnO). With the S3 sensor array it is possible to detect the presence of the microorganisms. This is accomplished through the detection of certain organic volatile compounds (VOCs) produced during the metabolic activities of the microorganisms. In some cases, it is even possible to identify a specific species among a group of microorganisms [60].

The instrument was also provided with the auto-sampler headspace system HT280 (HTA srl, Brescia, Italy), supporting a 40 loading sites carousel and a shaking oven to equilibrate the sample headspace at 40 °C for 10 min. The headspace (2 mL) was adsorbed and injected into the carrier flow at a speed of 4 mL/min. In order to have a reproducible sensor baseline, synthetic chromatographic air was used. A gas chromatography injector (kept at 40 °C to prevent any condensation) was adapted to produce a continuous flow rate of 10 mL/min of air. The time needed to recover the baseline was 28 min. The data analysis was carried out by means of principal component analysis (PCA).
